# Trafficking in and to the primary cilium

**DOI:** 10.1186/2046-2530-1-4

**Published:** 2012-04-25

**Authors:** Yi-Chun Hsiao, Karina Tuz, Russell J Ferland

**Affiliations:** 1 Department of Biology, Rensselaer Polytechnic Institute, Troy, NY 12180, USA; 2 Albany Medical College, Center for Neuropharmacology and Neuroscience, Albany, NY 12208, USA; 3 Department of Neurology, Albany Medical College, Albany, NY 12208, USA

**Keywords:** Primary cilium, Trafficking, Ciliopathies, Intraflagellar transport, Ciliary signaling

## Abstract

Polarized vesicle trafficking is mediated by small GTPase proteins, such as Rabs and Arls/Arfs. These proteins have essential roles in maintaining normal cellular function, in part, through regulating intracellular trafficking. Moreover, these families of proteins have recently been implicated in the formation and function of the primary cilium. The primary cilium, which is found on almost every cell type in vertebrates, is an organelle that protrudes from the surface of the cell and functions as a signaling center. Interestingly, it has recently been linked to a variety of human diseases, collectively referred to as ciliopathies. The primary cilium has an exceptionally high density of receptors on its membrane that are important for sensing and transducing extracellular stimuli. Moreover, the primary cilium serves as a separate cellular compartment from the cytosol, providing for unique spatial and temporal regulation of signaling molecules to initiate downstream events. Thus, functional primary cilia are essential for normal signal transduction. Rabs and Arls/Arfs play critical roles in early cilia formation but are also needed for maintenance of ciliary function through their coordination with intraflagellar transport (IFT), a specialized trafficking system in primary cilia. IFT in cilia is pivotal for the proper movement of proteins into and out of this highly regulated organelle. In this review article, we explore the involvement of polarized vesicular trafficking in cilia formation and function, and discuss how defects in these processes could subsequently lead to the abnormalities observed in ciliopathies.

## 

Primary cilia are evolutionarily conserved organelles projecting from the plasma membrane in almost every vertebrate cell. In general, primary cilia serve as sensors through which cells receive signals from light, chemical, or mechanical stimuli [1]. Moreover, the involvement of primary cilia in several signaling pathways important for development and tissue homeostasis (including the Sonic hedgehog and Wnt signaling pathways) has attracted much interest and stimulated extensive studies on this ancient cellular structure [2-6]. A functional primary cilium is required to properly activate primary cilia-mediated cellular signaling. Therefore, any defects in primary cilia could lead to cellular dysfunction. Indeed, abnormalities in primary cilia have been linked to a constellation of phenotypically and genetically overlapping human diseases, which include Bardet-Biedl syndrome, Joubert syndrome, Meckel-Gruber syndrome, nephronophthisis and Sensenbrenner syndrome; all now collectively known as ciliopathies [7-10]. The clinical manifestations of these disorders can include brain malformations, skeletal abnormalities, retinal degeneration, and cystic kidney disease.

The formation and function of primary cilia are tightly regulated by polarized vesicle trafficking, not only to the primary cilium, but also in coordination with trafficking throughout the entire cell [5]. Although bioinformatic, proteomic and genetic studies have suggested that more than a thousand proteins can be localized at the primary cilium, it is still unclear why and how these proteins work together in this specialized cellular compartment [11-15]. Therefore, studying the formation and function of the primary cilium, through investigations into the function of these ciliary proteins, will help to elucidate the pathophysiological mechanisms responsible for causing the ciliopathies.

## Structure and function of the primary cilium

**Figure 1:**
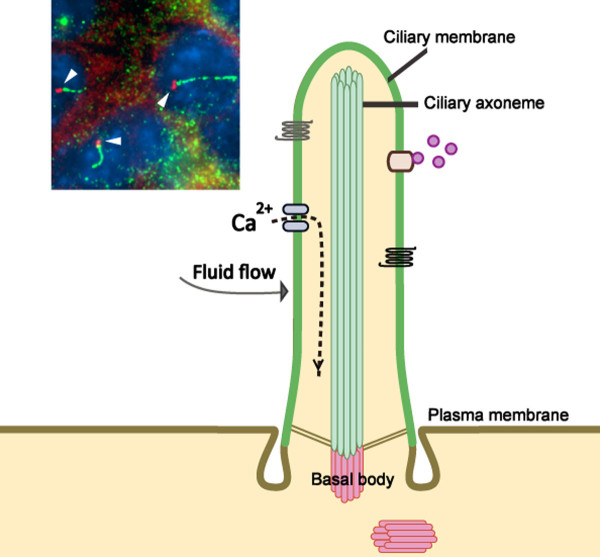
**Structure of the primary cilium**. The core structure of primary cilia is composed of microtubule bundles (ciliary axoneme) extending from the basal body, a microtubule-based structure derived from the mother centriole. The ciliary membrane is continuous with the plasma membrane, but contains a unique protein composition, such as channels and receptors. Thus, primary cilia can function as a sensory organelle for receiving and transducing extracellular stimuli into cells, such as fluid flow or via signaling molecules. The inset image is of ciliated murine inner medullary collecting duct (IMCD3) cells in which the basal body is labeled with a γ-tubulin antibody (red) and the primary cilium is marked by an Ift88 antibody (green).

Cilia are categorized into two classes: motile and non-motile. Motile cilia, such as tracheal cilia, can be numerous on a cell surface and have the prominent function of moving mucus and fluids, but are not the focus of this review. This review focuses on non-motile cilia, also referred to more commonly as primary cilia, which are solitary and mainly serve as a sensory organelle for the cell. Importantly, most primary cilia are non-motile, except for those present in the ventral nodes of vertebrates [1,16]. Primary cilia are polarized structures protruding from the surface of the cell into the extracellular space and are present on almost every quiescent cell in the body. The ciliary axoneme, which is composed of microtubule bundles, is the core structure of the cilium [1]. For primary cilia, the ciliary axoneme consists of a radial array of nine doublet microtubules with no central pair of singlet microtubules, and therefore is called a "9 + 0" configuration. The microtubule axoneme is nucleated at the basal body just beneath the plasma membrane (Figure 1). The basal body is a cytosolic microtubule organizing center that is derived from the mother centriole [1]. All of these structural components of the primary cilium are necessary for the proper formation and function of this signaling structure.

Primary cilia are enriched with receptors and provide a separate highly regulated compartment in which signaling events are conveyed from the extracellular space into the cell. Sensing the extracellular environment is a major function of primary cilia. For instance, the ciliated cells of the retina (photoreceptors) and the olfactory system (olfactory sensory neurons) receive and transduce the stimuli of light and odorants to the cells, respectively [17,18]. Primary cilia on the epithelial cells of kidney tubules act as mechanosensors for sensing fluid flow resulting in increased intracellular calcium signaling [19-21].

Recently, a vital role for primary cilia in signaling pathways important for embryonic development and tissue homeostasis has been identified. While the sonic hedgehog (Shh) pathway has been long known as a critical component of neural tube closure and organ patterning during embryonic development, it was only recently discovered that primary cilia are necessary for this signaling [2,6,22]. In mammalian cells when Shh is absent, the Shh receptor, Patched-1 (Ptch1), is localized at the base of the primary cilium and suppresses the activity of another transmembrane protein, Smoothened (Smo). Upon Shh stimulation, Smo is relieved from Ptch1 suppression and translocates to the primary cilium, while Ptch1 is removed from the primary cilium. This translocation of Smo to the primary cilium, after Shh stimulation, is required for activation of Shh-mediated downstream signaling [23-25].

Importantly, the integrity of the primary cilium and the proper functioning of the intraflagellar transport (IFT) system (the specialized transport system in the primary cilium) are both required for proper Shh signaling in mice [6,26]. For example, *Ift172 *deficient mouse embryos display abnormal brain development resulting from defects in cilia formation, consequently leading to reduced Shh signaling [27]. In addition to Shh signaling, primary cilia are also critical for Wnt signaling [3,28,29], calcium signaling [30,31], growth factor signaling [32-34], G-protein coupled receptor signaling [35,36], and receptor tyrosine kinase signaling [37]. Interestingly, the range of different types of signaling associated with cilia is ever expanding as more receptors and proteins are identified at the primary cilium. Although it is unclear why receptors are enriched at the primary cilium as compared to the plasma membrane or why signaling mechanisms are concentrated at the primary cilium, it is clear that this unique and separate cellular compartment makes primary cilia an unusual structure for receiving and transducing a variety of signals.

## Mechanisms for sorting ciliary bound proteins to the primary cilium

To date, there have been no studies which have shown that proteins can be produced in the cilium. Therefore, the unique density of proteins and receptors on the membrane around the ciliary axoneme were targeted there through a highly regulated importing process [25]. Trafficking of proteins from the cytosol and Golgi to the primary cilium, and for moving proteins along the ciliary axoneme, is regulated by polarized vesicle trafficking and intraflagellar transport, respectively. Detailed mechanisms of these two trafficking systems will be discussed later.

The transition zone (TZ), a region adjacent to the basal body, provides a selective barrier that can exclude vesicles, impede diffusion of proteins and lipids between compartments (cytosolic/plasma membrane versus ciliary), and control the entry and exit of proteins from the primary cilium [38-40]. The TZ is composed of transitional fibers (TFs), the ciliary necklace and Y-links. TFs anchor the basal body to the proximal ciliary membrane, forming a pinwheel-like structure whose protein composition is unknown. The ciliary necklace is distal to the TFs and consists of rows of protein particles around the ciliary membrane at the base of the cilium. Y-links connect axonemal microtubules to the membrane at the ciliary necklace [41]. Whereas TZ components include CC2D2A, MKS1, MKS3, MKS5, MKS6, MKS1 related-1, MKS1 related-2, NPHP1, NPHP4, Septin 2, Tctn1, and Tctn3 [40-44], the Y-links are associated with CEP290 localization [39]. Importantly, different components perform different functions at this region. For instance, while CEP20 functions as a gate keeper, controlling cilia protein composition by restricting the entrance of non-ciliary proteins, CC2D2A facilitates the entry of ciliary proteins through a role in vesicle trafficking [39,42].

To aid in this trafficking, ciliary proteins contain a targeting sequence enabling it to efficiently localize at the primary cilium. To date, different targeting sequences have been identified: rhodopsin possesses a C-terminal targeting sequence with a VxPx motif; polycystin-2 has an N-terminal RVxP motif; and polycystin-1 harbors a C-terminal targeting sequence, KVHPSST [45,46]. This has led to the suggestion of a VxP motif as a generic ciliary targeting sequence (CTS). However, cystin contains a N-terminal CTS with a AxEGG motif [47], whereas the G-protein coupled receptors, Sstr3, Htr6, and Mchr1, present an Ax(S/A)xQ targeting sequence in their third intracellular loop [48]. Overall, these data indicate that there is no unique consensus CTS and further suggest that there is more than one molecular mechanism involved in the recognition of such sequences.

Palmitoylation and myristoylation are post-translational modifications that provide a point of membrane association and have been identified as a requirement for some ciliary proteins in their proper targeting to the cilium. For example, the targeting sequence for rhodopsin contains two cysteine residues that are palmitoylated and which are necessary for rod outer segment targeting [49]. The same is true for fibrocystin, in which its CTS has three palmitoylated cysteine residues [50]. Lastly, cystin is myristoylated at its G2 residue and it has been shown that this acylation is required for the proper localization of cystin to the ciliary membrane [47]. Therefore, the sorting of ciliary targeted proteins to the primary cilium not only occurs through CTSs, but can also be accomplished through post-translational modifications. It is these modifications that provide the sorting information for proteins resulting in their proper trafficking to the specialized membrane microdomains of the primary cilium.

Targeting of proteins to the primary cilium also involves regulation of proteins under the control of the GDP-GTP cycle. Rab8, one such protein, interacts with the CTS of fibrocystin, as suggested by a study using a GFP fusion of the fibrocystin CTS [50]. This GFP-CTS localizes to primary cilia in cells expressing Flag-Rab8 and Flag-Rab8Q67L (a constitutively active form of Rab8a having decreased GTPase activity, thereby maintaining the protein in the GTP bound state), but not Flag-Rab8T22N (a dominant negative form of Rab8 having a higher affinity for GDP than GTP) expressing cells. Rab8T22N bound more GFP-CTS than either the wild type or the Q67L mutant, suggesting that activation of Rab8 results in the release of the GFP-CTS from Rab8. Therefore, the regulation of Rab8 activity controls the localization of fibrocystin through its CTS. Whether other ciliary localizing proteins containing CTSs interact with Rabs through their CTS, remains to be determined.

A mechanism for NPHP3 ciliary targeting has been partially described in which myristoylation of its G2 residue and regulation of GTPase activity are required. That is, NPHP3, when it is myristoylated, binds to UNC119 (HRG4). The phenylalanine residues lining the hydrophobic β-sandwich in UCN119 contribute to this NPHP3 binding. This complex is trafficked through an unknown mechanism to the primary cilium. In the cilium, ARL3-GTP binds to its effector, UNC119, releasing myristoylated NPHP3 into the cilium or ciliary membrane. RP2 then activates ARL3 GTPase activity, releasing UNC119 to reset the cycle [51]. These and other studies clearly indicate that protein sorting to the primary cilium is a tightly regulated process that may involve multiple molecular mechanisms of protein-protein recognition and protein-membrane interaction, in which the GDP-GTP state of these proteins is fundamental.

## Functional primary cilia depend on polarized trafficking and intraflagellar transport

**Figure 2:**
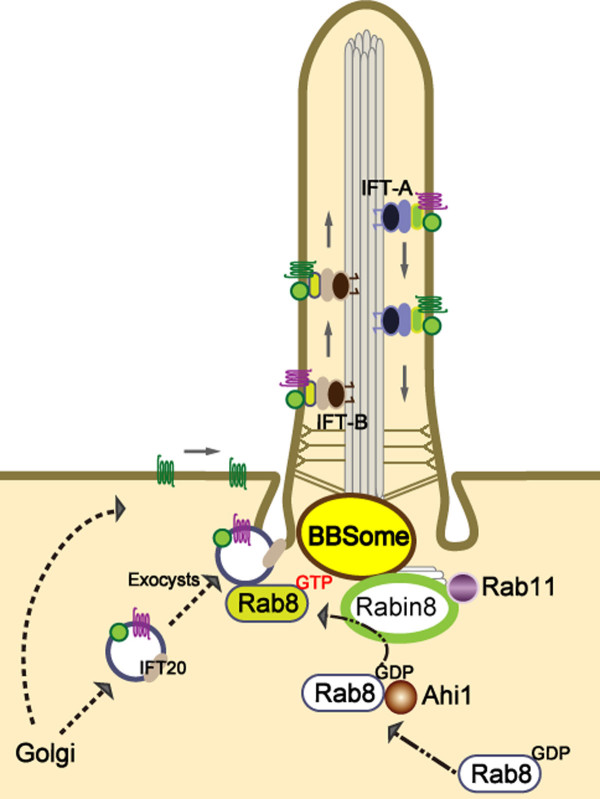
**Polarized vesicle trafficking mediates the formation and function of primary cilia**. Enriched expression of receptors and ion channels on the ciliary membrane make the primary cilium a specialized organelle for receiving and transducing extracellular stimuli into cells. Proteins synthesized from the Golgi move to the primary cilium through polarized vesicle trafficking utilizing microtubule networks (not shown). For instance, vesicles carrying ciliary proteins leave the Golgi and move toward the basal body of the primary cilium. These vesicles can either be delivered to the surface plasma membrane and then the protein cargo moves to the ciliary membrane or they can be trafficked toward the basal body through Rab proteins, IFT20, or exocysts. Entry of protein cargo to the cilium is regulated by active forms of Rab8, a master modulator for the ciliary protein trafficking. Rab8 is recruited to the basal body of primary cilium, possibly mediated by Ahi1. The activities of Rab8 are then regulated by Rabin8, and its activity and basal body localization is modulated by the BBSome and Rab11. Once proteins are transported into the primary cilium, the IFT system continues the trafficking of these proteins or membrane receptors up and down along the ciliary axoneme. IFT-B (anterograde) and IFT-A (retrograde) are protein complexes associated with the molecular motors, kinesin-2 and cytoplasmic dynein, respectively.

Polarized vesicle trafficking is a highly regulated delivery system in cells for transporting proteins and vesicles to their proper destinations in order to perform and maintain normal cell function (Figure 2). In most cases, the primary cilium is formed at the end of the cell cycle, after cell polarity is established. At the early stage of cilium formation (ciliogenesis), a vesicle derived from the Golgi encapsulates the distal end of the mother centriole (the origin of the basal body) as it migrates toward the apical plasma membrane. After docking of the basal body, the primary cilium elongates as the axoneme extends. Additional vesicles carry ciliary membrane proteins to the cilium, which then fuse to the plasma membrane where the cilium originates [52]. Thus, ciliogenesis requires axoneme assembly, membrane biogenesis, and a proper compartmentalization of ciliary proteins in coordination with polarized vesicle trafficking [1,53].

### Polarized vesicular trafficking in ciliogenesis and ciliary function

Polarized vesicle trafficking is a specialized cellular transport mechanism used to deliver proteins and membranes to their proper cellular compartments (Figure 2). Perturbations in this process can have significant impacts on normal cell function [54,55]. Emerging evidence has shown an important link between polarized vesicle trafficking and the proper formation and function of the primary cilium. Not only have Golgi-derived vesicles been implicated in early ciliogenesis, but the constant trafficking of vesicles through the post-Golgi to the primary cilium has been shown to be crucial for normal ciliary structure and function [56,57]. Polarized vesicle trafficking is mediated by Rabs along with Arf/Arl members of the Ras superfamiliy of small GTPases, which assist in the recruitment of vesicle coating complexes during vesicle budding, docking and fusion of vesicles [58,59]. Additionally, the essential roles of these small GTPase proteins in the formation and function of primary cilia have been demonstrated in several studies (Table 1) [2,60,61]. The activities of Rabs and Arf/Arl proteins are regulated by their nucleotide binding status. GDP-bound Rab proteins, usually referred to as inactive forms, are switched to the GTP-bound active state through catalyzation by guanine nucleotide exchange factors (GEFs). Then, GTP-bound Rabs can modulate several cellular events through activation of their downstream effectors, including vesicle sorting proteins, tethering proteins, kinases, phosphatases and motor proteins. Conversion of active GTP-bound Rabs back to inactive GDP-bound Rabs is through GTP hydrolysis by GTPase activating proteins (GAPs). The correct membrane targeting of Rab proteins is crucial and regulated by its specific GDP dissociation inhibitors (GDIs) and membrane-bound GDI displacement factors (GDFs) [59].

**Table 1:** Genes/proteins involved in primary cilia trafficking

ITF particle/component	IFT20, Elipsa
Rab GTPases	Rab5, Rab6, Rab8, Rab10, Rab11, Rab23
Arf/Arl GTPases	ARF4, ARL3, ARL6, ARL13, ARL13b
Guanosyl nucleotide exchange factor	Rabin8 (Rab8)
GTPase activating protein	RP2 (ARL3), ASAP1 (Arf4)
Rab effectors	Rabaptin5 (Rab5), FIP3 (Rab11)
Clathrin adaptor	AP-1
Exocyst	Sec3, Sec5, Sec6, Sec8, Sec10, Sec15, Exo70, Exo84
BBSome	BBS1, BBS2, BBS4, BBS5, BBS7, BBS8, BBS9, BBIP10
TRAPPII complex	TRAPPC1, TRAPPC2, TRAPPC3, TRAPPC4, TRAPPC5, TRAPPC6A, TRAPPC6B, TRAPPC9, TRAPPC10

#### Arl proteins in ciliary trafficking

Arl6 is exclusively expressed in ciliated organisms [62], and its human ortholog ARL6, encoded by *BBS3*, was the first identified small GTPase protein that was linked to the human ciliopathy, Bardet-Biedl syndrome (BBS) [63]. This rare inherited disorder features dysfunction in multiple organ systems, leading to retinal dystrophy, obesity, renal disease and cognitive impairments [64]. The abnormalities associated with the loss of BBS3 in BBS are thought to arise due to dysfunctional primary cilia [63]. That is, Arl6 is localized at the distal end of the basal body, near or at the TFs, a region of the ciliary compartment that controls the entry of proteins into the primary cilium [65]. Arl6 functions as a recruiter of the BBSome (a basal body localizing complex composed of seven BBS disease proteins) to the basal body and as a regulator of the function of the BBSome in ciliary protein trafficking [60,66]. ARL13B is another small GTPase protein connected to the human ciliopathy, Joubert syndrome, an inherited neurodevelopmental disorder with midbrain-hindbrain malformations, retinal dystrophy and, occasionally, nephronophthisis [67]. Deletion of *Arl13b *in mice leads to the deformation of primary cilia and defective neuronal tube development due to hyperactive Shh signaling [2,68]. Work in *Caenorhabditis elegans *has demonstrated that the formation of the IFT-A and IFT-B subcomplex is critical for building the ciliary structure and that the coordination of Arl13 and Arl3 is required for stabilizing this subcomplex [69-71].

#### Rab small GTPases

Rab GTPases are the largest family of small GTPases, and their function and distinct localization at intracellular membranes has been studied extensively [59]. However, the distribution of several Rab GTPases at the primary cilium was only recently discovered. Disruption of the ciliary localization or the activities of these Rab GTPases are associated with several ciliopathies due to impairments in cilium formation and function [72]. For example, Rab8 is a critical modulator for the formation and the function of primary cilia in addition to having an important role in vesicular trafficking between the trans-Golgi and the basolateral membrane [53,73]. As mentioned previously, rhodopsin is present at the outer segments of photoreceptor cells. Rab8 is, in part, responsible for delivering rhodopsin-bearing post-Golgi vesicles close to the base of the photoreceptor connecting cilium, where these vesicles fuse, and rhodopsin is then transported through the connecting cilium to the outer segment [74]. Rab8 mutations that interfere with the GTP/GDP cycle of the protein have been utilized to test its role on ciliogenesis. Work in *Xenopus laevis *has shown that Rab8T22N (a dominant negative form of Rab8) and Rab8Q67L (a constitutively active form of Rab8) expression disrupts rhodopsin trafficking and leads to retinal degeneration, a common occurrence in human ciliopathies [75]. Expression of Rab8T22N in RPE or IMCD3 cells was shown to inhibit cilia formation, whereas expression of Rab8Q67L promoted ciliogenesis in these cell types [50,60]. In *Danio rerio*, injection of Rab8T22N results in abnormalities in Kupffer's Vesicle (embryonic ciliated structure similar to the node of mammals), presumably through defects in cilia [60]. This suggests that the activity of Rab8 is critical for the biogenesis of ciliary membrane [60]. Indeed, studies of BBS and other human ciliopathies demonstrate that the ciliary localization and activity of Rab8 is critical for cilium formation and function.

The integrity of the BBSome is necessary for the regulation of Rab8 activity and only the constitutively active form of Rab8 is able to enter the primary cilium [60]. Therefore, mutations of any BBS disease protein in the BBSome would consequently affect the activity of Rab8 at the primary cilium. Moreover, it has also shown that the activity of Rab8 can also be regulated by the retinitis pigmentosa GTPase regulator (RPGR), a ciliary protein implicated in X-linked retinitis pigmentosa [76]. This suggests that there are multiple regulatory mechanisms for Rab8 activity that can be used in ciliated cells or even in different cell types.

The targeting of Rab8 to the basal body is essential for the function of Rab8 at the primary cilium. The basal body localizing protein AHI1, when mutated, can cause the human ciliopathy Joubert syndrome [77-79], and is required for recruiting Rab8 to the basal body [80,81]. Knockdown of *Ahi1 *expression in IMCD3 cells impairs ciliogenesis and results in the loss of Rab8 localization to the basal body. Interestingly, these phenotypes cannot be rescued by overexpressing a constitutively activated form of Rab8, indicating the requirement for Ahi1 in the proper localization for Rab8 [80]. Also, mice with a targeted deletion of *Ahi1 *develop retinal degeneration with an accumulation of rhodopsin in the photoreceptor inner segments, possibly due to a decrease in the levels of photoreceptor Rab8 expression [82]. Overall, this implies that the localization of Ahi1 at the basal body is required for the ciliary targeting of Rab8.

Moreover, the central role of Rab8 in vesicle targeting to the primary cilium has been supported by many studies showing an association of Rab8 with other proteins involved in vesicular trafficking to the primary cilium [50,83,84]. For instance, studies in *C. elegans *have shown that Rab8 genetically interacts with Rabaptin5, an endocytosis regulator, and through which it forms a complex with Elipsa, an IFT particle polypeptide binding to Ift20, thereby regulating protein trafficking to the primary cilium [84]. In ciliated sensory neurons in *C. elegans*, coordination of Rab8 with the vesicle coating complex, AP-1, was found to be necessary for sorting and trafficking of ciliary membrane [83]. In addition to regulating proteins that participate in vesicle trafficking, Rab8 is also directly involved in targeting proteins to the primary cilium through its interaction with CTSs in these proteins. Fibrocystin, a ciliary protein which is encoded by a gene implicated in autosomal recessive polycystic kidney disease, fails to traffic to the primary cilium when Rab8 activity is inhibited in IMCD3 cells [50]. Overall, the data discussed above clearly support a fundamental role for Rab8 in modulating vesicle targeting to the primary cilium.

In addition to Rab8, several other Rab GTPases have also been identified at the primary cilium and have been shown to function in different ciliary trafficking pathways or processes. A key regulator of endosome recycling, Rab11, is enriched at the base of primary cilia, and compromising the function of Rab11 in cells abolishes ciliogenesis [85]. The mechanism by which Rab11 is involved in cilia formation is through the recruitment of Rabin8, a Rab8 specific GEF, to the centrosome, thereby stimulating centrosomal Rab8 activity during early cilium formation [86]. Rab11 also participates in ciliary targeting by recognizing conserved ciliary targeting sequences in ciliary proteins [87].

Our knowledge of the function of various Rab small GTPases in ciliary trafficking is growing through the use of ciliary fluorescence recovery after photo-bleaching (FRAP) [88]. Consistent with previous studies that have shown a direct role of Rab8 in controlling proteins entering the primary cilium, ciliary FRAP experiments have demonstrated that cells expressing a dominant negative Rab8 exhibit a slower fluorescent recovery rate for the GFP-bound ciliary proteins, Smo and Kim1 (an apical membrane receptor), indicating a disruption of ciliary anterograde transport. In addition, results of FRAP experiments in cells expressing a dominant negative Rab5 exhibited a slower fluorescent recovery rate only for Kim1 indicating that there are likely multiple pathways for delivering ciliary proteins. Conversely, inactivation of Rab23, a protein that is essential for ciliogenesis, does not block the entry of proteins into the primary cilium, but has effects on their recycling to the primary cilium. This interpretation was based on the finding that while these cells had a normal fluorescent recovery rate, they had had an increase in the fluorescence intensity recovered in the cilium supporting a role for Rab23 in protein recycling. However, expression of a dominant negative Rab23 specifically influences only Smo and not Kim1. As a result, Rab23 is necessary for maintaining the level of Smo at the primary cilium through its fundamental role in Smo recycling, providing a mechanism for Rab23 that is proposed to act as a negative regulator in Shh signaling [89]. This further supports the essential role for primary cilia in Shh signal transduction.

#### Exocysts

The exocyst is a conserved octameric protein complex consisting of Sec3, Sec5, Sec6, Sec8, Sec10, Sec15, Exo70, and Exo84, and it is involved in basolateral protein sorting and membrane trafficking in cells [90]. The integrity of the exocyst is essential for exocytosis in yeast [91] and for the ability of Madin-Darby canine kidney (MDCK) cells to form cysts in culture [92]. The distribution of exocysts in cells is not only at cell-cell junctions, but also at the primary cilium in polarized cells [12,93]. Compromising the function of exocysts by knockdown of Sec10 results in the formation of shortened primary cilia, along with reduced levels of Sec8, Exo70, and Ift88 [94]. This suggests a central role for Sec10 in stabilizing the exocyst complex and possibly a role of Sec10 in trafficking Ift88 to the primary cilium. The involvement of the exocyst in early ciliogenesis has been suggested by an association of Sec8 and dishevelled (Dvl), a protein involved in the planar cell polarity (PCP) signaling pathway [95]. During basal body docking in multi-ciliated cells, Sec8 cannot co-localize with the basal body when Dvl is knocked down. The exocyst, labeled by Sec6 and Sec8, that is localized at the base of the photoreceptor-connecting cilium, co-localizes with Rab8 at the fusion sites of rhodopsin carrying vesicles, implicating a role for the exocyst as a vesicle tether at cilia [96]. In addition to Rab8, an association of exocysts with Rab10 has also been demonstrated by a direct interaction and co-localization of Sec8 and Rab10 at the basal body of a nascent primary cilium [97]. This interaction of Rab10 and Sec8 suggests that coordination of the exocyst and Rab10 is important in mediating the biogenesis of the ciliary membrane [97]. Lastly, the binding of Sec15 and Rab11 was found to regulate the function of exocysts in basolateral-to-apical transcytosis [98]. Although this study does not describe whether primary cilia were defective when Rab11 binding to Sec15 was disrupted, it does suggest another pathway through which the exocyst could be involved in regulating the primary cilium.

#### Microtubules

EB1 and EB3 are microtubule plus end tracking proteins that localize to the base of the primary cilium in human fibroblasts and RPE cells [99]. EB1 also localizes to the basal body in the green algae, *Chlamydomonas *[100]. Both proteins are important for cilia formation, likely through a role in microtubule minus end-basal body anchoring activity, since the microtubule array anchored at the basal body is disorganized in EB1 and EB3 knockdown cells having aberrant vesicle accumulation and impaired ciliogenesis. These results demonstrate the relevance of microtubules in anchoring to the basal body thereby providing a 'roadmap' for the targeting of vesicles carrying ciliary proteins to the vicinity of the basal body where they are exocytosed [99,101].

### Intraflagellar transport in ciliogenesis and ciliary function

Transport of axonemal precursors and several ciliary membrane proteins to the ciliary tip is mediated by IFT, a process which moves cargos up and down along the ciliary axoneme [102,103]. Given that IFT is highly conserved among organisms with cilia and flagella, much of our current knowledge about IFT comes from work on *Chlamydomonas*. Newly synthesized ciliary proteins made in the cell body need to be transported into the cilium. This occurs through the association of these ciliary proteins with the IFT complex B (IFT-B), which then moves its cargo toward the cilium tip (anterograde transport) under the power of the molecular motor, kinesin-2 [104]. Conversely, retrograde transport delivers proteins back to the base of the primary cilium through the IFT complex A (IFT-A) using the motor protein, dynein-2 [56,105]. At least twenty proteins have been identified to date as components of the IFT-A and IFT-B complexes in ciliated mammalian cells [22,106]. The crucial role of IFT in the formation and function of primary cilia has been elucidated by genetic studies utilizing IFT mutants [6,107-109]. For instance, mice with deletions of the gene encoding Ift88/Polaris, a component of IFT-B, develop polycystic kidney disease (PKD), hepatic fibrosis, and *situs inversus*, resulting from malformed or absent primary cilia [107,110]. Not only is proper functioning of IFT critical for cilia formation, but it is also necessary for its maintenance. Tubulin turns over steadily on the flagella tips in *Chlamydomonas*, but resorption does not occur since IFT continuously delivers tubulin to the microtubule ends, thereby regulating flagella length [111]. Similarly, the outer segments of photoreceptors contain stacks of membrane discs, which receive and transduce light signals. These membrane discs undergo rapid turnover leading to the replacement of entire outer segments every two weeks [112]. Importantly, the link between the outer segment and the inner segment of the photoreceptor is through a connecting cilium, the analog to the TZ, and is critical for the proper maintenance of photoreceptor outer segments [113,114]. Therefore, functional IFT is essential for transporting newly synthesized proteins and membrane to the outer segment through the connecting cilium [115,116]. In support of this, mice with conditionally deleted *Kif3A *have an abnormal distribution of rhodopsin in the photoreceptor inner segments; an aberrant localization since rhodopsin is rapidly transported from the inner segments to the outer segments via the connecting cilium. Interestingly, it is this aberrant localization of rhodopsin in the cell body that results in the subsequent loss of photoreceptors due to increased reactive oxygen species accumulation [117]. Similar phenotypes are also observed in Ift88/Polaris mutant mice and other IFT mutant animals [110,118,119].

IFT is also important for cilium-dependent signaling, with the Shh pathway as the most striking example [120,121]. Studies of IFT mutant mouse models have suggested that functional IFT is essential for trafficking of Shh signaling components in the primary cilium and for activating downstream signaling [6,107,122]. For instance, patterning of the neural tube and formation of the limbs are determined by a gradient of Shh signaling. Mice with deletion of *Ift88 *fail to form primary cilia and display phenotypes resembling mice with reduced Shh signaling, such as loss of ventral neuronal cell types and polydactyly [120,123]. Shh phenotypes have also been observed in other anterograde IFT mutant mouse models, such as with *Ift172 *and *Kif3A *knockouts [6,124]. Furthermore, mutant mice with defective retrograde IFT usually exhibit phenotypes reminiscent of excessive Shh signaling [22,125,126]. Disruption of the retrograde IFT-A complex in mice by deleting one of its components, *Ift122*, results in defective ciliogenesis and uncontrolled Shh signaling. More specifically, *Ift122 *knockout mouse embryos have a ventrolateral expansion of motoneurons and ectrosyndactyly (absence of digits), all consistent with an excess in Shh signaling [126]. However, given the diversity of phenotypes that are observed in IFT mutants and the involvement of different tissues, this would suggest that the regulatory role of IFT proteins in Shh signaling may be more complicated. Case in point, *Ift88 *deficient mice display an abnormal neural tube and defective limb development resulting from decreased Shh signaling, but these mutant mice also show ectopic molar tooth development due to hyperactive Shh signaling [127]. Lastly, the function of IFT in Shh signaling varies depending on the model system used. Reducing Ift88 expression in zebrafish disrupts ciliogenesis but does not affect Shh signaling, unlike the defective Shh signaling observed in *Ift88 *knockout mice [128,129]. Also, in *Drosophila*, primary cilia are not required for proper Shh signaling and Ift *Drosophila *mutants do not have defective Shh signaling [62,130,131]. Whether the regulatory roles that IFT and primary cilia have in mediating Shh signal transduction are restricted to mammals or more generally to vertebrates still remains to be elucidated.

While IFT particles principally transport cargos along the primary cilium, they can occasionally carry vesicles containing ciliary proteins to the primary cilium from the Golgi. That is the case for Ift20, which is a component of IFT-B and is also localized at the Golgi. Like other IFT mutants, reduced expression of Ift20 in cells impairs cilia formation [132]; moreover, disrupting the Golgi localization of Ift20 through knockdown of GMAP20, a Golgi anchoring protein for IFT20, results in a loss of the membrane protein, polycystin-2, at the primary cilium [133]. These results implicate the involvement of IFT20 in the trafficking and sorting of ciliary proteins between the Golgi and the primary cilium.

## Ciliopathies result from impaired ciliary trafficking

Polarized vesicle trafficking is the foundation for functional primary cilia, and disruption at any step impairs the formation and function of primary cilia. Indeed, studies of the molecular mechanisms responsible for human ciliopathies demonstrate that ciliary function is compromised when vesicle trafficking is disrupted. BBS has been extensively studied as a ciliopathy model to understand how its mutant proteins affect the primary cilium and consequently lead to the observed clinical pathologies. An individual with BBS displays malfunctions in multiple organ systems, including retinal degeneration, cognitive dysfunction, obesity, polydactyly, and cystic kidney disease. Like most ciliopathies, BBS is a genetically heterogeneous disease that can be caused by mutations in fourteen genes [134]. Seven BBS proteins (BBS1, BBS2, BBS4, BBS5, BBS7, BBS8, and BBS9) and one novel protein BBIP10 compose the BBSome. The other BBS proteins are not considered as part of the BBSome itself, but are involved in regulating either the assembly or function of the BBSome [135,136]. Therefore, mutations in any of the BBS proteins in this network could affect ciliary function and result in similar clinical phenotypes in BBS [137]. To date, the major function for the BBSome appears to be in modulating ciliary trafficking. Recruitment of the BBSome to the basal body is regulated by Arl6, which is encoded by BBS3 as mentioned earlier [66]. The localization of the BBSome to the basal body is essential for modulating the activity and the ciliary entry of Rab8, through its association with Rabin8 via BBS1 [60]. Moreover, the BBSome also participates in the sorting and trafficking of ciliary membrane proteins. Depletion of *Bbs4 *in mouse neurons causes a lack of ciliary localization of the somatostatin receptor 3 (Sstr3) and the melanin-concentrating hormone receptor 1 (MchR1) [35]. This is due to a direct interaction of the BBSome and the intracellular loop 3 of Sstr3, which contains its ciliary targeting sequence [66]. Given that the composition of the BBSome resembles the structure of the canonical vesicle coat complexes and its ability to recruit lipids, it has been suggested that the BBSome may function as the ciliary membrane protein sorting machinery at the primary cilium [66].

In addition to the BBS proteins, several ciliopathy-related proteins have been linked to ciliary trafficking. *CEP290 *encodes for a centrosomal protein, mutations of which have been linked to several ciliopathies, including BBS [138], Joubert syndrome [79,139], nephronophthisis, and Leber Congenital Amaurosis [140]. It has been proposed that CEP290 may function as a gatekeeper for the primary cilium to control ciliary trafficking [141]. Electron microscopic examination of flagellated *Chlamydomonas *has shown that cep290 is associated with the flagellar membrane and the microtubules at the transition zone of flagella, a structure providing a diffusion barrier for selective cilium transport. Also, *cep290 *depletion in *Chlamydomonas *results in malformation of the flagella along with aberrant ciliary protein composition in isolated flagella, indicative of a loss of a flagellar diffusion barrier. While there was no effect on anterograde IFT in cep290 mutants, a slight effect on retrograde IFT was observed [39]. These results indicate that the function of CEP290 in ciliary trafficking is more likely at the level of entry of proteins into the primary cilium. In further support, mutations in *CEP290 *are also associated with BBS [138]; however, CEP290 is not part of the BBSome. Therefore, the mechanism for how CEP290 regulates ciliary trafficking through the BBSome remains unknown.

In addition to a function of Ahi1 in modulating Wnt signaling [142-144], recent studies have now shown that Ahi1 is also linked to polarized vesicle trafficking in ciliated cells [80,82]. Knockdown of *Ahi1* expression in IMCD3 cells not only reduces cilium formation, but also affects the endocytosis of chlorea toxin A and transferrin receptors. Also, *Ahi1* knockdown cells exhibit abnormal Golgi structure and location suggesting that polarized vesicle trafficking has been severely affected with loss of Ahi1 [80]. However, additional studies are needed examining whether this abnormal Golgi structure and non-apical positioning occurs in cells from individuals with *AHI1 *mutations. Consistent with the results from IMCD3 cells, neurons isolated from mice with a targeted deletion of *Ahi1 *fail to form primary cilia [80]. However, work in another *Ahi1 *knockout mouse has shown that primary cilia are found in normal numbers, but clearly have signaling defects [142-144]. This discrepancy in ciliogenesis, and also in the presence of cystic kidney disease [143] (unpublished observations), may be accounted for by the background strain of the mice used or whether the gene was targeted conditionally or not. Studies showing significant decreases in ciliogenesis in *Ahi1 *knockout mice were on a pure inbred line using a traditional knockout strategy, whereas the studies not showing differences in cilia formation were on a mixed background and were conditional knockouts. In support of the background strain hypothesis, all *Ahi1 *knockout mice on a pure C57BL6/J or C3H/HeJ background die within 12 hours following birth; however, the same knockout mice on a FVB/NJ or BALB/cJ background have a significant increase in survival, even into adulthood (unpublished observations). Importantly, this is not a result of maternal behavior. These observations suggest that there are likely modifying genes in the various inbred strains of mice that may account for the differences in ciliogenesis observed in different knockout mice. This raises an important consideration when comparing results from knockout mice on different genetic backgrounds and provides a difficult caveat for interpreting results demonstrating the impact of genes on cilia formation and function.

The finding of ciliopathy-disease proteins modulating ciliary protein trafficking clearly indicates the importance of these processes and how disruptions in ciliary trafficking could result in the developmental abnormalities and organ malfunction observed in ciliopathies. Moreover, given the genetic heterogeneity and phenotypic variability displayed in the ciliopathies, this would suggest that the regulatory mechanisms in primary cilia are mediated by multiple proteins, and are likely cell-type dependent. Areas of new interest in the field, with important implications for understanding the function of primary cilia, are how ciliary proteins leave and are recycled back to the primary cilium. This new avenue of research could result in therapeutic strategies for the treatment of the ciliopathies. Primary cilia, originally thought of as vestigial structures, actually have dynamic and complex vesicle trafficking regulation through which signaling can be properly performed. Through the study of the unique roles of primary cilia in cellular function, we hope to understand better and possibly treat the vast array of clinical symptoms that result when cilia are dysfunctional.

## Abbreviations

BBS: Bardet-Biedl syndrome; CTS: Ciliary targeting sequence; Dvl: Disheveled; FRAP: Fluorescence recovery after photo-bleaching; GAP: GTPase activating protein; GDI: GDP dissociation inhibitor; GDF: GDP dissociation inhibitor displacement factor; GEF: Guanine nucleotide exchange factor; IFT: Intraflagellar transport; IMCD: Inner medullary collecting duct; MDCK: Madin-Darby canine kidney; PCP: Planar cell polarity; PKD: Polycystic kidney disease; RPE: Retinal pigment epithelium; RPGR: Retinitis pigmentosa GTPase regulator; TFs: Transition fibers; TZ: Transition zone.

## Competing interests

The authors declare that they have no competing interests.

## Authors' contributions

YH wrote the first draft. KT made substantial changes to the manuscript. RJF commented, rewrote, and edited the draft, in addition to commenting on and editing the drafts. All authors read and approved the manuscript.
